# Uniform Nickel Vanadate (Ni_3_V_2_O_8_) Nanowire Arrays Organized by Ultrathin Nanosheets with Enhanced Lithium Storage Properties

**DOI:** 10.1038/srep20826

**Published:** 2016-02-10

**Authors:** Chang Wang, Dong Fang, Hong’en Wang, Yunhe Cao, Weilin Xu, Xiaoqing Liu, Zhiping Luo, Guangzhong Li, Ming Jiang, Chuanxi Xiong

**Affiliations:** 1 Key Lab of Green Processing and Functional Textils of New Textile Materials, Ministry of Education, College of Material Science and Engineering, Wuhan Textile University, Wuhan 430070, China; 2 School of Materials Science and Engineering, Wuhan University of Technology, Wuhan 430070, China; 3 Department of Chemistry and Physics, Fayetteville State University, Fayetteville, USA; 4 State Key Laboratory of Porous Metal Material, Northwest Institute for Non-ferrous Metal Research, Xi’an 710000, China

## Abstract

Development of three-dimensional nano-architectures on current collectors has emerged as an effective strategy for enhancing rate capability and cycling stability of the electrodes. Herein, a novel type of Ni_3_V_2_O_8_ nanowires, organized by ultrathin hierarchical nanosheets (less than 5 nm) on Ti foil, has been obtained by a two-step hydrothermal synthesis method. Studies on structural and thermal properties of the as-prepared Ni_3_V_2_O_8_ nanowire arrays are carried out and their morphology has changed obviously in the following heat treatment at 300 and 500 °C. As an electrode material for lithium ion batteries, the unique configuration of Ni_3_V_2_O_8_ nanowires presents enhanced capacitance, satisfying rate capability and good cycling stability. The reversible capacity of the as-prepared Ni_3_V_2_O_8_ nanowire arrays reaches 969.72 mAh·g^−1^ with a coulombic efficiency over 99% at 500 mA·g^−1^ after 500 cycles.

Energy conversion and storage is undoubtedly one of the greatest challenges in today’s world^1^. Lithium ion batteries (LIBs) are deemed among the best choices owing to their high specific energy and long cycle life in portable electronic consumer devices, electric vehicles, and large-scale electricity storage in intelligent grids^2^,^3^,^4^. It has led the extensive research efforts on the development of electrode materials with higher specific capacity. In particular, the materials that store lithium ions (Li^+^ ions) through conversion reactions (such as MnO_2_^5^, Co_3_O_4_^6^, V_2_O_5_^7^) or alloying reactions (such as Si^8^, Sn^1^) have been suggested as promising alternative materials due to their intrinsically high specific capacity. However, one drawback exists for commercially available LIBs electrode materials due to the intrinsic diffusivity of Li^+^ ions in the solid state (about 10^*−*8^ cm^2^ s^−1^), which unavoidably limits the charge/discharge performance^9^,^10^. Further, these materials typically undergo significant volume change during lithiation and delithiation due to the large Li atoms uptake in the structure and accompanying phase transformation. Approaches for enhancing ion/electron transport kinetics and accommodate strain induced by volume change in LIBs include coating electrolytically active material with a conductive layer^11^,^12^,^13^. Designing the electrode materials with nanoscale features is an alternative useful method because nanostructures can help to reduce the diffusion length for Li^+^ ions during the charge/discharge process and increase the interfacial contact area between electrode and electrolyte, thus leading to significantly enhanced specific power density and energy density as compared with non-nanostructure materials^14^,^15^,^16^,^17^,^18^,^19^. Hierarchical structure materials with at least one dimension on the nanometer length scale (hierarchical nanostructures) can combine desirable bulk-material properties (such as structural stability and high tap density) with size-tunable functional properties for the construction of electrochemical energy storage devices (LIBs and supercapacitors)^20^,^21^.

Nickel oxide (NiO), a base transition metal oxide enriched in nature resources with environmental benignity, has been considered as one of the fascinated electrode materials for lithium storage^22^,^23^,^24^,^25^. The main challenges to implement NiO based anodes are their low electronic conductivity and large volume change during lithium insertion and extraction as mentioned above. To address these issues, binary metal oxides, such as NiMn_2_O_4_^26^, and NiCo_2_O_4_^27^, have also been proposed as anode materials to improve their electrochemical performances. In this case, the binary metal oxides have much higher electrical conductivity and electrochemical performances than single oxide^27^,^28^. The higher electronic conductivity is favorable for the rapid transfer of electrons in an electrode. Recently, transition metal vanadates (MV_x_O_y_), which are related to V_2_O_5_ with tunable oxidation states (V^5+^, V^4+^ and V^3+^), have received increasing attention for potential applications in various fields due to their layered structure, unique physical, chemical, and electrical properties^7^,^29^. The electrode cycling stability of pure vanadium oxides has been greatly improved by addition of M (=Li, Fe, Cr, or Na) ions into these host vanadium oxides, such as LiV_3_O_8_^30^, FeVO_4_^31^, Cr_0.11_V_2_O_5.16_^32^, Na_5_V_12_O_32_^33^. These additional M ions were arranged to form pillars between the vanadium oxide layers and thus stabilized the structure during the Li^+^ insertion/extraction^33^,^34^.

In the present work, we present an efficient two-step hydrothermal synthesis method to synthesize Ni_3_V_2_O_8_ nanowire arrays organized by ultrathin nanosheets. To our knowledge, Ni_3_V_2_O_8_ structures such as nanowires or nanosheets have not been reported previously. Morphological evolution and phase transformations of Ni_3_V_2_O_8_ nanowire arrays during the calcinations process are studied by field emission scanning electron microscopy (FE-SEM), transmission electron microscopy (TEM), and X-ray diffraction microscopy (XRD). Due to the unique properties of these nanowire arrays, such as high surface areas, crystallinity, good conductivity and direct growth on conductive substrates, they have potential applications in LIBs, chemical sensing, electrochemical or photocatalysis, field emission, and electrochromic devices^35^,^36^. As an electrode material for LIBs, the as-grown Ni_3_V_2_O_8_ nanowires on Ti foil present outstanding energy storage properties.

## Results and Discussion

**Figure 1:**
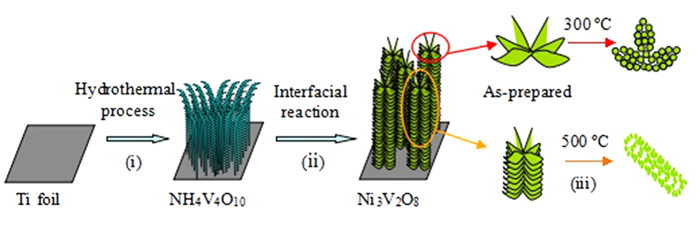
Schematic illustration of the formation process of Ni_3_V_2_O_8_ nanowire arrays: (**i**) formation of NH_4_V_4_O_10_ nanowire arrays on the surface of the Ti foil, (**ii**) further growth of Ni_3_V_2_O_8_ nanowire arrays organized by ultrathin nanosheets, and (**iii**) formation of porous structure through calcinations.

The general electrode fabrication protocol is illustrated in Fig. 1. First, NH_4_V_4_O_10_ nanowire arrays are grown directly on a Ti substrate via a facile modified hydrothermal process. The obtained almost crystalline NH_4_V_4_O_10_ nanowire arrays are then subjected to impregnation with C_4_H_6_O_4_Ni aqueous solution for reaction, which leads to obtain Ni_3_V_2_O_8_ nanowire arrays. This is enlightened by the fact that Ni_3_V_2_O_8_ can be produced by the green reaction between NH_4_V_4_O_10_ and C_4_H_6_O_4_Ni, in the absence of any acid or base (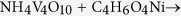

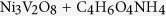
). Here, the ordered NH_4_V_4_O_10_ nanowires obtained in the first step are purposely designed as an interfacial reactive template to grow Ni_3_V_2_O_8_ nanowire arrays (step ii). For comparison, the as-prepared Ni_3_V_2_O_8_ nanowire arrays are annealed in air, interesting, the nanosheets on the nanowires transfer to nanoparticles after heat treatment at 300 °C, and the nanowires change to porous or tubular structure after annealing at 500 °C.

The corresponding XRD patterns provide verification of the detailed structural and phase information used to index the as-obtained samples. The XRD patterns of NH_4_V_4_O_10_ and Ni_3_V_2_O_8_ after heat treatments at 300 and 500 °C are presented in Fig. S1a. The as-prepared Ni_3_V_2_O_8_ obtained after hydrothermal reaction exhibits an orthorhombic structure. After heat treatment at 500 °C, the sample shows a highly crystalline nature and the whole diffraction peaks can be indexed to Ni_3_V_2_O_8_ orthorhombic phase (JCPDS card No. 70-1394)^37^. No characteristic peaks, related to NiO, TiO_2_, NH_4_V_4_O_10_, or V_2_O_5_, are detected in the produced material, indicating pure Ni_3_V_2_O_8_ nanowires on the surface of Ti substrate. Comparing the curves in Fig. S1a, it is noted that XRD peaks of the as-prepared Ni_3_V_2_O_8_ are broadened. A variety of factors can be contributed to the width of a diffraction peak besides instrumental effects and crystallite size, and the most important factors are usually inhomogeneous strain and crystal lattice imperfections, or crystallinity^38^,^39^. From the crystal effect on diffraction by the Scherrer equation^40^, the crystal size of the as-prepared Ni_3_V_2_O_8_ is smaller than that of the annealed samples (see Table S1 in supporting information). Further, the XRD peaks (2*θ*) shift to higher values with increasing the treatment temperature. A small change in peak shift does not necessarily mean phase change. Solid solution, be it substitutional or interstitial, can cause a shift in the x-ray diffraction peaks. Another two factors which contribute to peak shift are residual stress and defects in the material, because these two factors also can deform the lattice, causing peak shift^41^. Figure S1b shows the unit cell consisting of nickel, vanadium and oxygen atoms, presenting the mixed valence oxides that crystallize in the orthorhombic system with lattice constants: *a* = 5.936 Å, *b* = 11.420 Å, and *c* = 8.240 Å. It also clearly displays the detailed structure which all nickel ions are the arrays of edge-shared NiO_6_ octahedra forming Kagome-like layers, and the layers are separated by VO_4_ tetrahedra, resulting in a peculiar Kagome-staircase geometry^42^.

X-ray photoelectron spectroscopy (XPS) (see Fig. S2 in the Supplementary Information) is used to demonstrate the structure and purity of the sample. A wide-range survey XPS spectrum of the as-prepared sample reveals the presence peaks of C 1s (at ∼284.6 eV), V 2p (at ∼517.1 eV), O 1s (at ∼531.9 eV) and Ni 2p (at ∼855.8 eV) (Fig. S2a). The C 1s peak at 284.6 eV is used as a reference binding energy for calibration. After annealing up to 300 °C, the XPS spectrum in Fig. S2b is similar to that of the as-prepared Ni_3_V_2_O_8_. The peak deconvolution and fittings are carried out using Gaussian-Lorentzian shaped peaks based on the Shirley background correction. Two typical peaks centered at 855.9 and 873.8 eV are observed (Fig. S2c), corresponding to spin-orbit peaks of the Ni 2p_3/2_ and Ni 2p_1/2_ of Ni_3_V_2_O_8_, respectively. Meanwhile, two satellite lines associated with Ni 2p also appear. From the binding energies of Ni 2p main lines and the splitting due to the spin-orbit coupling, besides the energy gaps separating the main lines and satellite peaks, it indicates that Ni (II) and Ni (III) cations together exist in Ni_3_V_2_O_8_ structures^43^. The high resolution scan of the V 2p core levels is performed in Fig. S2d, in which the peak position of V 2p_3/2_ is fitted using a Shirley function^44^. The V species exist with a close binding energy value of V^5+^ 2p_3/2_ (516.4–517.4 eV) and V^4+^ 2p_3/2_ (515.4–515.7 eV)^45^,^46^. Therefore, the peak fitted at 517.0 eV can be mainly ascribed to V^5+^ 2p_3/2_, whereas that at 515.6 eV is assigned to V^4+^ 2p_3/2_. Thus, the electron couples of Ni^3+^/Ni^2+^ and V^5+^/V^4+^ are coexisted in the orthorhombic Ni_3_V_2_O_8_ structures, where the total atomic ratio of Ni and V elements is about 3:2, corresponding to the molecular formula of Ni_3_V_2_O_8._

**Figure 2:**
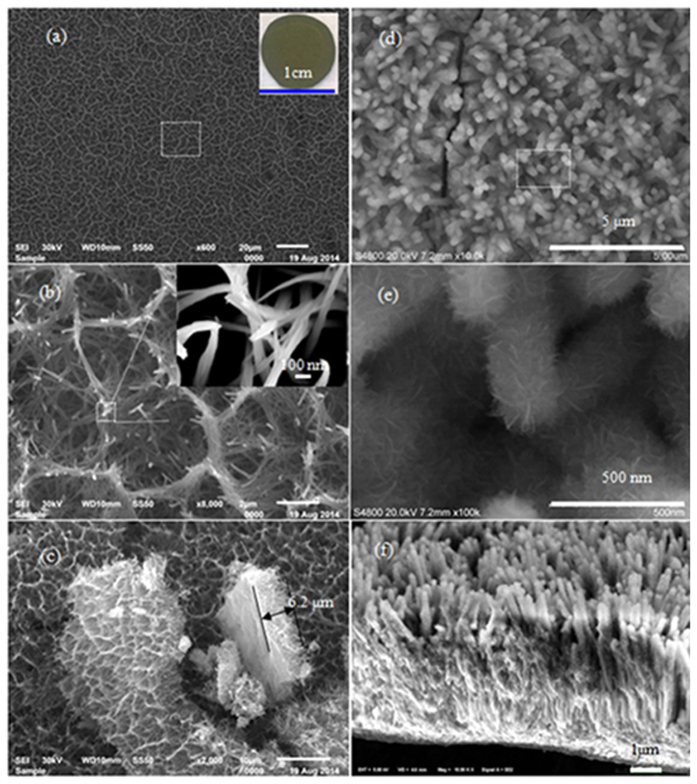
(**a**–**c**) The FE-SEM images of the NH_4_V_4_O_10_ nanowire arrays and (**d**–**f**) the as-prepared Ni_3_V_2_O_8_ nanowire arrays; (**a**,**b**,**d**,**e**) the top-view FE-SEM images; and (**c**,**f**) the cross-sectional FE-SEM images. The inset in (**a**) shows a digital image of the NH_4_V_4_O_10_ nanowire arrays on Ti foil.

The morphology structures of the as-synthesized NH_4_V_4_O_10_ precursor and Ni_3_V_2_O_8_ nanowire arrays are characterized by the FE-SEM technique, as shown in Fig. 2. Figure 2a,b show the top-view FE-SEM images of the NH_4_V_4_O_10_ nanowire arrays. The precursor nanowires are distributed uniformly and adhered firmly to the surface of the Ti substrate. The inset in Fig. 2a is a digital image of the nanowire arrays on a Ti foil. The NH_4_V_4_O_10_ nanowire arrays grow vertically of the substrate and can reach up to 6.2 μm, according to the cross-section FE-SEM view in Fig. 2c. After the second hydrothermal reaction, the surface of the aligned nanowires becomes rough, and the nanowires are changed to Ni_3_V_2_O_8_ arrays, as shown in Fig. 2d–f, which are homogeneously aligned and separated apart. The length, diameter, and inter-wire space of Ni_3_V_2_O_8_ nanowire arrays are about 6.8 μm (Fig. 2f), 321 nm (Fig. 2e), and 100 nm (Fig. 2e), respectively. Meanwhile, the second hydrothermal reaction of NH_4_V_4_O_10_ and C_4_H_6_O_4_Ni with different reaction time is studied and the results are shown in Fig. S3. After 1 min reaction, the surfaces of the NH_4_V_4_O_10_ nanowires become rough and the diameter of the nanowires are larger (~130 nm). 10 minute later, the diameter of the nanowires is about 260 nm and the nanowires are coated with nanosheets. When the reaction time is further extended to 2 h, the diameter is even larger and the wires are composed of nanosheets. The corresponding schematic plot of the Ni_3_V_2_O_8_ formation process is presented in Fig. S3e. During the extended reaction time, the NH_4_V_4_O_10_ nanowires react with nickel salt from their surfaces to inners.

**Figure 3:**
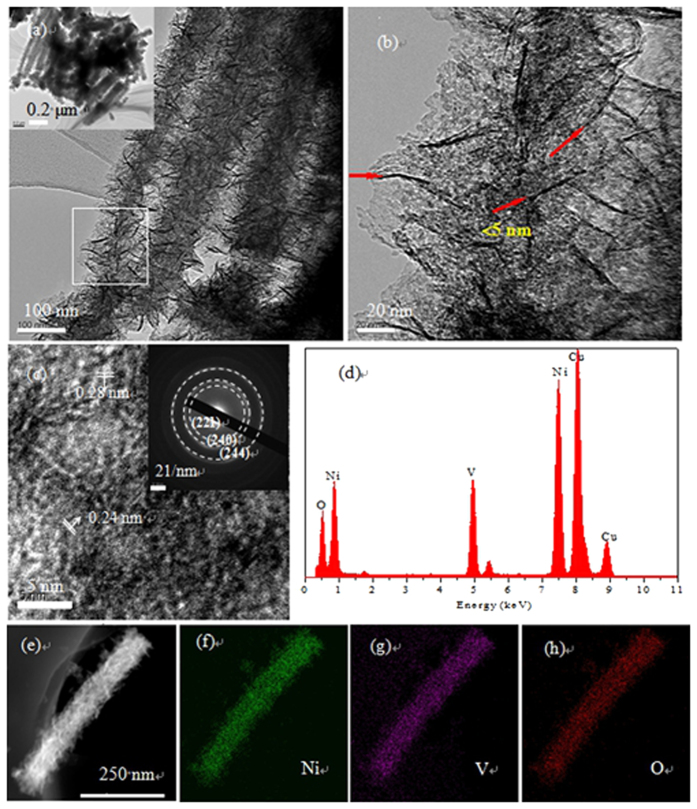
Ni_3_V_2_O_8_ nanowire arrays scratched from Ti foil: (**a**,**b**) TEM image at different magnifications; (**c**) HR-TEM image (inset: SAED pattern); and (**d**–**h**) EDS maps of a single nanowire.

The morphology of the as-prepared Ni_3_V_2_O_8_ nanowire arrays is further researched by TEM. Figure 3a shows a TEM image of an individual hybrid nanostructure in which Ni_3_V_2_O_8_ nanowire arrays uniformly organized by ultrathin nanosheets. In addition, the thickness of the nanosheets is less than 5 nm from the contrast in the TEM image (Fig. 3b). The HR-TEM image shown in Fig. 3c reveals fringes with interplanar spacing of 0.24 nm and 0.28 nm, respectively, corresponding to the (131) and (115) plane of the orthorhombic Ni_3_V_2_O_8_ (JCPDS card No. 70-1394). The selected area electron diffraction (SAED) pattern (the inset in Fig. 3c) indicates the polycrystalline nature of the Ni_3_V_2_O_8_ nanosheets and the cycles can be readily indexed to (221), (240) and (244) crystal planes of the orthorhombic Ni_3_V_2_O_8_ phase. An energy dispersive X-ray spectrometer (EDS) spectrum (Fig. 3d) shows that the atomic ratio of Ni/V/O is approximately 23.7/15.8/60.5, which is close to the ratio of Ni/V/O in the Ni_3_V_2_O_8_ formula. The Cu signal in the spectrum is from Cu grid used during TEM characterization. Locations of the different elements are illustrated by element mapping: Ni (Fig. 3f), V (Fig. 3g), and O (Fig. 3h). It shows the entire nanowire is composed of Ni, V, and O elements, suggesting the formation of homogeneous Ni_3_V_2_O_8_ nanowires.

After calcination at 300 °C, Ni_3_V_2_O_8_ nanowires nearly keep their structure as shown in Fig. S4a. While the detail views in Fig. S4b,c present that the Ni_3_V_2_O_8_ nanosheets transform into nanoparticles, which is further demonstrated by the TEM images as shown in Fig. S4d,e. The HR-TEM image shown in Fig. S4f reveals the interplanar spacing of 0.15 nm, corresponding to the (244) plane of the orthorhombic Ni_3_V_2_O_8_. The SAED pattern (inset of Fig. S4f) also confirms that the nanoparticles are made of orthorhombic Ni_3_V_2_O_8_. During heat treatment at 500 °C, the nanosheets on the wire will be curved to tubes (Fig. S5). As a result, the nanowire arrays organized by ultrathin nanosheets change to porous or tubular structures assembled by particles. In order to explain reason why the solid Ni_3_V_2_O_8_ nanowires become tubular ones, the as-prepared samples were annealed at 500 °C with different time (Fig. S6). After 20 min, the nanosheets changed to wire-like structure. At 40 or 60 min, the wire-like structure further assembled to particle-like structure and this phenomenon is more obvious after 2 h. The corresponding schematic plot of the Ni_3_V_2_O_8_ formation process is presented in Fig. S6e. The nanosheets assemble from their edge to central and forms wire-like structure. Further, the wire agglomerates from its two ends to central and form particle-like structure. The as-prepared Ni_3_V_2_O_8_ nanosheets are assembled from single axles (NH_4_V_4_O_10_ nanowires) (Fig. S3 in the support information), and therefore, the particles yielded from these nanosheets also surrounded axially on the symcenters. The particles are at the geometric centrals of the original nanosheets, and if the particle size is ignored, the inner diameter of the tubular wires is about 1/2 radial length of the nanosheets.

The aligned Ni_3_V_2_O_8_ nanowire arrays on Ti foil are evaluated as an electrode material for LIBs in view of their many appealing structural features. Fig. S7 shows the cyclic voltammogram (CV) plots of the first three cycles at a sweep rate of 0.1 mV s^−1^ within a potential window of 0.1–4 V (vs. Li/Li^+^ ions). It can be seen that two pairs of redox peaks appear at 2.13/1.19 V and 2.77/2.41 V (vs. Li/Li^+^ ions) for the as-prepared NH_4_V_4_O_10_ nanowire arrays (Fig. S7a). The CV curves of the as-prepared Ni_3_V_2_O_8_ nanowire arrays (Fig. S7b) and that annealed at at 300 °C (Fig. S7c) have a similar feature. In the first cathodic scan, a strong peak at 0.35–0.50 V can be detected corresponding to Faradaic redox reactions of the decomposition of active materials Ni_3_V_2_O_8_ to Ni^0^ 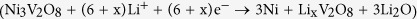. In the positive going potential scan, two obvious anodic peaks at 1.48 V and 2.44 V are observed, associated with the oxidation of Ni and the decomposition of Li_x_V_2_O_8_, respectively^47^. Here, an irreversible capacity loss in the first cycle is caused by amorphous Li_x_V_2_O_8_ producing and solid electrolyte interface (SEI) film forming. In the subsequent cycles, a peak at 0.55 V is observed corresponding to a reversible reaction of 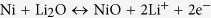, and another peak at 1.52 V relates to the equation of 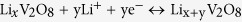. In the subsequent cycles, the third cycle curve coincides with the second cycle well, indicating that the electrode reactions become more reversible.

**Figure 4:**
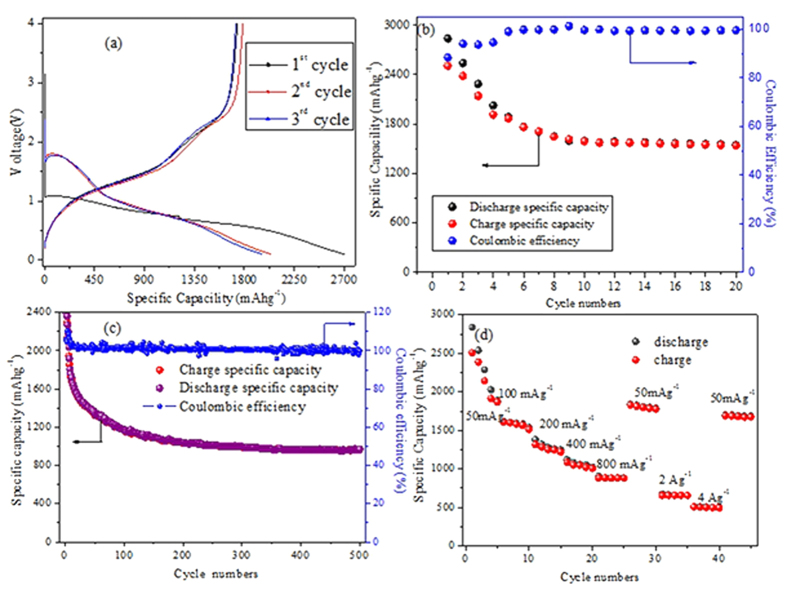
Electrochemical lithium storage properties of as-prepared Ni_3_V_2_O_8_ nanowire arrays on Ti foil: (**a**) the initial three charge/discharge voltage profiles at a constant current density of 50 mA∙g^-1^; cycling performance at a current density of (**b**) 50 mA∙g^-1^ or (**c**) 500 mA∙g^-1^; and (**d**) rate performance at various current densities from 50 mA∙g^-1^ to 4 A∙g^-1^.

The electrochemical performances of the as-prepared Ni_3_V_2_O_8_ nanowire arrays for LIBs are also evaluated by galvanostatic discharge/charge test. All of the capacities in Fig. 4 are based on the whole mass of Ni_3_V_2_O_8_ nanowire arrays with a cut-off voltage of 0.1–4 V (versus Li/Li^+^ ions). Figure 4a shows the initial three discharge/charge curves of the Ni_3_V_2_O_8_ nanowire arrays at 50 mA·g^−1^, revealing the obvious platforms during the lithiation and delithiation processes. In the charge curve, we can see two potential plateaus located at 1.44 and 2.50 V, which correspond to the two oxidation peaks in the CV curve (Fig. S7). Another two potential plateaus are found in the discharge curve located at 0.57 and 1.62 V, agreeing with those reduction peaks in the CV curve. The initial discharge and charge capacities of the Ni_3_V_2_O_8_ electrode are up to 2837.07 and 1706.76 mAh·g^−1^, respectively. A specific capacity of 1545.53 mAh·g^−1^ can be remained after 20 cycles at 50 mA·g^−1^ (Fig. 4b). Further, the reversible capacity reaches 969.72 mAh·g^−1^ with a coulombic efficiency over 99% at 500 mA·g^−1^ after 500 cycles (Fig. 4c), demonstrating the excellent cycling stability of the as-prepared Ni_3_V_2_O_8_ nanowire arrays electrode. Impressively, our hierarchical Ni_3_V_2_O_8_ nanowire arrays exhibit remarkably high-rate capability as shown in Fig. 4c. For each step, 5 cycles are measured to evaluate the rate performance. The average discharge capacities of the electrode at 50, 100, 200, 400, 800 mA·g^−1^, 2 and 4 A·g^−1^ are 2315.01, 1585.52, 1296.73, 1065.34, 887.12, 662.26 and 502.81 mAh·g^−1^, respectively (shown in Fig. 4d). It also reveals that after cycling at high-rate, the capacity can be recovered once the current density is returned back to 50 mA·g^−1^, implying excellent reversibility and rate capability. The multilevel hierarchical architecture based on nanosheet subunits and the hollow structure among the sheets offers a robust and porous framework, large electrode/electrolyte contact area, and reduced Li^+^ ions diffusion distance, all of which benefit the electrochemical reaction kinetics in the electrode. On the other hand, such architecture can better withstand huge volume variation associated with repeated lithiation/delithiation process.

Figure S8a gives the cycle ability of the samples obtained at varieties temperatures (the as-prepared Ni_3_V_2_O_8_ nanowire arrays and that annealed at 300 or 500 °C). It clearly shows that the as-prepared Ni_3_V_2_O_8_ nanowire arrays can provide much higher capacities than that of their corresponding annealed samples, which is attributed to following reasons. After annealing at 300 or 500 °C, the Ni_3_V_2_O_8_ nanosheets transform partly or totally into higher crystalline nanoparticles instead of the pure nanosheet structure. The self-assembled nanoparticals electrodes typically suffer from poor electrical conductivity due to the large interparticle spacing maintained, which will reduce the amount of active material taking part in electrochemical reaction and lower the lithium storage capacity^48^.

For comparison, the cycling performances of Ni_3_V_2_O_8_ nanowire arrays grown or pasted on Ti foil electrodes at a same current density (300 mA·g^−1^) are shown in the Fig. S8b. It is observed that the Ni_3_V_2_O_8_ nanowire arrays grown on the Ti foil electrode delivers a reversible capacity up to 1200.24 mAh·g^−1^ after 20 cycles. While, the reversible specific capacity of the Ni_3_V_2_O_8_ nanowire arrays pasted on the Ti foil electrode reduces from 1505.82 mAh·g^−1^ to 911.51 mAh·g^−1^ during 20 cycles. It indicates that Ni_3_V_2_O_8_ nanowire arrays grown on the Ti foil can make use of the advantages of special reinforcement properties to maintain the cycling reversibility and higher retention specific capacity. Electrochemical impedance spectroscopy (EIS) test is carried out to further understand the advantage of the Ni_3_V_2_O_8_ nanowire arrays grown on the current collector (Ti foil) (Fig. S8c). Ni_3_V_2_O_8_ nanowire arrays grown or pasted on Ti foil electrodes have the similar shapes of Nyquist plots, composed of a high-frequency semicircle and a long low-frequency subsequent 45° line. The black lines are the fitting curves by using the equivalent circuit, which is shown in the inset. It is made up of a parallel combination of a constant phase element (CPE), charge transfer resistance (Rp), Weber impedence (W), and solution resistance (Rs). The perfect semicircle was hardly achieved in this real system, thus, a CPE is used instead of a double-layer capacitance (C_dl_). As shown in Table S2, the Rs of the Ni_3_V_2_O_8_ electrodes changes from 6.16 Ω (grown on Ti foil) to 15.70 Ω (pasted on Ti foil), manifesting a good conductivity of the electrolyte and the lower ionic and electronic resistance of the nanowires grown on Ti foil. A schematic in Fig. S8d presents charge storage mechanism of the Ni_3_V_2_O_8_ nanowire array electrode. In the discharge process, electrons will pass through the contact point of the nanowires and the titanium substrate, and then up along the nanowires. Li^+^ ions can migrate in the electrolyte, reach the electrode, and take part in the electrochemical reaction with Ni_3_V_2_O_8_. In the charge process, the movements of the Li^+^ ions and electrons are reverse. By growing directly from the NH_4_V_4_O_10_ nanowire scaffold, Ni_3_V_2_O_8_ nanowire arrays organized by ultrathin nanosheets are well separated, making them fully available to the Li^+^ ions in the electrolyte. The directly grown nanowire arrays can ensure good mechanical adhesion and electrical connection to the current collector, which avoids extra contact resistance.

For comparation, a cut-off voltage of 0.1–3.0 V (versus Li/Li^+^) was selected during carrying out the electrochemical properties of the Ni_3_V_2_O_8_ nanowire arrays growing on Ti foil. The electrochemical properties are shown in Fig. S9. Figure S9a shows the CV plots of the first three cycles at a sweep rate of 0.1 mV s^−1^ within a potential window of 0.1–3.0 V (vs. Li/Li^+^) and the peak positions are similar to that obtained with a cut-off voltage of 0.1–4.0 V (versus Li/Li^+^). The initial three charge/discharge voltage profiles at a constant current density of 50 mA∙g^-1^ within a potential window of 0.1–3.0 V (vs. Li/Li^+^) are shown in Fig. S9b. Further, a specific capacity of 1389.57 mAh·g^−1^ is remained at the current densities of 50 mA·g^−1^ after 20 cycles (Fig. S9c) or 890.89 mAh·g^−1^ at the current densities of 500 mA·g^−1^ after 500 cycles (Fig. S9d). Further, the morphology stability of the sample after 50 cycles are tested and presented in Fig. S10. The overall wire structures can be generally retained, although the detailed nanosheet structures become somewhat diminished and thicken. This is understandable, as these nanosheets might be too thin and large to withstand the high-rate insertion/extraction of lithium ions over extended cycling^49^. Further, the volume expansion will occur after lithium ion insertion, therefore, the nanosheets broaden after galvanostatic charge-discharge cycling.

## Conclusion

In summary, we demonstrated, for the first time, a new type of Ni_3_V_2_O_8_ nanowire arrays organized by ultrathin nanosheets obtained by a two-step hydrothermal synthesis method on a Ti substrate. In virtue of the structural advantages, the obtained Ni_3_V_2_O_8_ nanowire arrays manifest excellent electrochemical properties as potential anode materials for LIBs in terms of high specific capacity, remarkable cycling ability, and superior rate capability. In our case, the superior electrochemical performances of Ni_3_V_2_O_8_ nanowire arrays result from the following factors: (i) uniform nanosized sheets, decreasing the electrochemical polarization; (ii) hierarchical structure, providing shortened Li^+^ ions/electron diffusion pathways and relieving to the volume changes during cycling; (iii) high structural stability, preventing the nanomaterials from dissolving into electrolyte; (iv) nanowire arrays on the current collector (Ti foil), enabling the active roles of nanowires in electrode reactions to benefit the transitions of ions and electrons. The present work indicates that the experimentally designed Ni_3_V_2_O_8_ nanowire arrays possesses great application potentials in high-performance energy storage devices, and this method may be extended for other transition metal oxides for the development of lithium ion batteries.

## Methods

### Synthesis Material

First, self supported NH_4_V_4_O_10_ nanowire arrays are prepared by a facile hydrothermal synthesis method. The solution is prepared by ultrasonic dissolving ammonium metavanadate (NH_4_VO_3_), oxalic acid (H_2_C_2_O_4_·2H_2_O), and hexamethylene tetramine (C_6_H_12_N_4_) in distilled water. The Ti foil as the substrate (1 cm in diameter) is immersed into the reaction solution. Then, this resulting solution is transferred into Teflon-lined stainless steel autoclave liners and heated to 150 °C for 40 min inside a conventional laboratory oven. Subsequently, the sample is rinsed with distilled water and dried at 50 °C to obtain a NH_4_V_4_O_10_ nanowire array on Ti foil. To fabricate Ni_3_V_2_O_8_ nanowire arrays, a piece of Ti substrate covered with the NH_4_V_4_O_10_ nanowire array is used as the scaffold for Ni_3_V_2_O_8_ nanoflake growth. The nickel acetate (C_4_H_6_O_4_Ni) aqueous solution is prepared by stirring dissolving C_4_H_6_O_4_Ni in distilled water. Then the NH_4_V_4_O_10_ nanowire arrays on Ti foil are put into a Teflon-lined stainless steel autoclave containing the nickel acetate aqueous solution as mentioned above at 120 °C for 2 h. Finally, the sample is removed, washed with distilled water and dried at 50 °C to obtain the Ni_3_V_2_O_8_ nanowire arrays organized by ultrathin nanosheets. For comparation, the as-prepared Ni_3_V_2_O_8_ nanowire arrays are annealed at 300 or 500 °C in air.

### Material Characterization

The samples are characterized by X-ray diffraction (XRD, RIGAKUD/Max-2550 with Cu Kα radiation), Energy dispersive X-ray spectrometer (EDS), Field emission scanning electron microscopy (FE-SEM, JSM-6700F at 160 kV), Transmission electron microscopy (TEM, Tecnai G220ST at 200 kV), and High resolution transmission electron microscopy (HR-TEM). The chemical composition of the sample is analyzed by X-ray photoelectron spectroscopy (XPS, Kα 1063, Thermo Fisher Scientific, UK).

### Electrochemical Characterization

Electrochemical analyses are performed using coin-type cells (CR2016) with lithium metal as the negative electrode, which are assembled in an argon-filled glove box at room temperature. The Ti substrates supported NH_4_V_4_O_10_ nanowire arrays, Ni_3_V_2_O_8_ (as-prepared) nanowire arrays and that annealed at 300 °C or 500 °C are directly used as the positive electrode without any binders or conducting additives. For comparation, the electrode slurry of the Ni_3_V_2_O_8_ nanowire powder is prepared by mixing the active material detached from Ti substrate using a knife, acetylene black (AB) and polyvinylidene fluoride (PVDF) with a weight ratio of 8:1:1. The resulting slurry is pasted onto a Ti foil, which is used as the current collector. The electrolyte is 1 M LiPF_6_ in ethylene carbonate (EC)/diethyl carbonate (DEC) (1:1, v/v). Cyclic voltammetry (CV) measurements are performed on a CHI660 electrochemical workstation in the voltage range of 0.1–4 V at a scanning rate of 0.1 mV s^−1^. The galvanostatic discharge/charge tests are conducted on a LAND battery program-control test system at different current densities in the voltage range of 0.1–4 V. Electrochemical impedance spectroscopy (EIS) is measured at an open voltage using a PGSTAT 302N electrochemical workstation (Autolab) in the frequency range of 0.01 Hz–5 kHz.

## Additional Information

**How to cite this article**: Wang, C. *et al*. Uniform Nickel Vanadate (Ni_3_V_2_O_8_) Nanowire Arrays Organized by Ultrathin Nanosheets with Enhanced Lithium Storage Properties. *Sci. Rep.* **6**, 20826; doi: 10.1038/srep20826 (2016).

## Supplementary Material

Supplementary Information
