# Supplementary material for: Uniform Nickel Vanadate (Ni3V2O8) Nanowire Arrays Organized by Ultrathin Nanosheets with Enhanced Lithium Storage Properties
Source: Sci Rep. 2016 Feb 10;6:20826. doi: 10.1038/srep20826 (PMC4748403; doi:10.1038/srep20826)
Supplement: Supplementary Information [file srep20826-s1.pdf]

# Supporting Information

## Uniform Nickel Vanadate ($Ni_3V_2O_8$) Nanowire Arrays Organized by Ultrathin Nanosheets with Enhanced Lithium Storage Properties

**Chang Wang[1], Dong Fang[1,*], Hong en Wang[2], Yunhe Cao[1], Zhiping Luo[3], Xiaoqing Liu[2], Guangzhong Li[4], Ming Jiang[1], Chuanxi Xiong[1], Weilin Xu[1,*]**

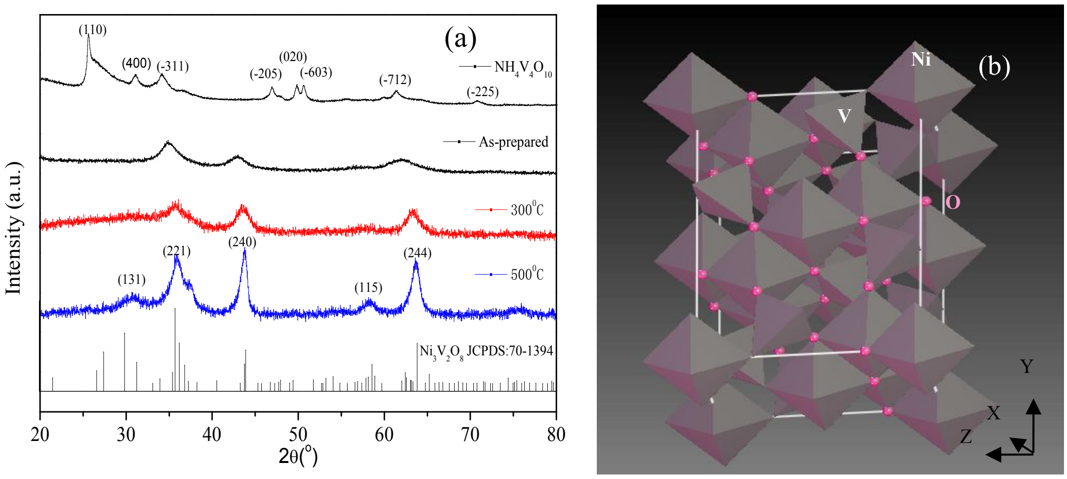


**Figure S1.** (a) XRD patterns of $NH_4V_4O_{10}$ nanowires and $Ni_3V_2O_8$ nanowires (annealing at different temperatures); and (b) structural model of orthorhombic $Ni_3V_2O_8$.

The crystallite sizes (D) were evaluated by the classical Scherrer equation (Equation S1),[1] the orthorhombic crystallite size (D) of $Ni_3V_2O_8$ nanowire arrays annealed at various temperatures was calculated from the X-ray diffraction peaks related to (221), (240) and (244) planes using the Scherrer equation (Equation (2)).[1]

$$D = \mathrm{k}\lambda / \beta \cos\theta \qquad (1)$$

$$D = k\lambda / (\beta \div 180 \times 3.14 \cos\theta) \qquad (2)$$

Where k is a shape constant, equal to 0.9, λ is the wavelength of the X-rays source used (Cu Kα= 0.15406 nm), and β and θ are Full width at half maximum (FWHM) and peak position (radians), respectively, determined after Voigt fitting. The results are presented in Table 1. It is evidenced the effect of annealing temperature for crystallite size of $Ni_3V_2O_8$. Overall, the crystallite size increases with the annealing temperature increases.

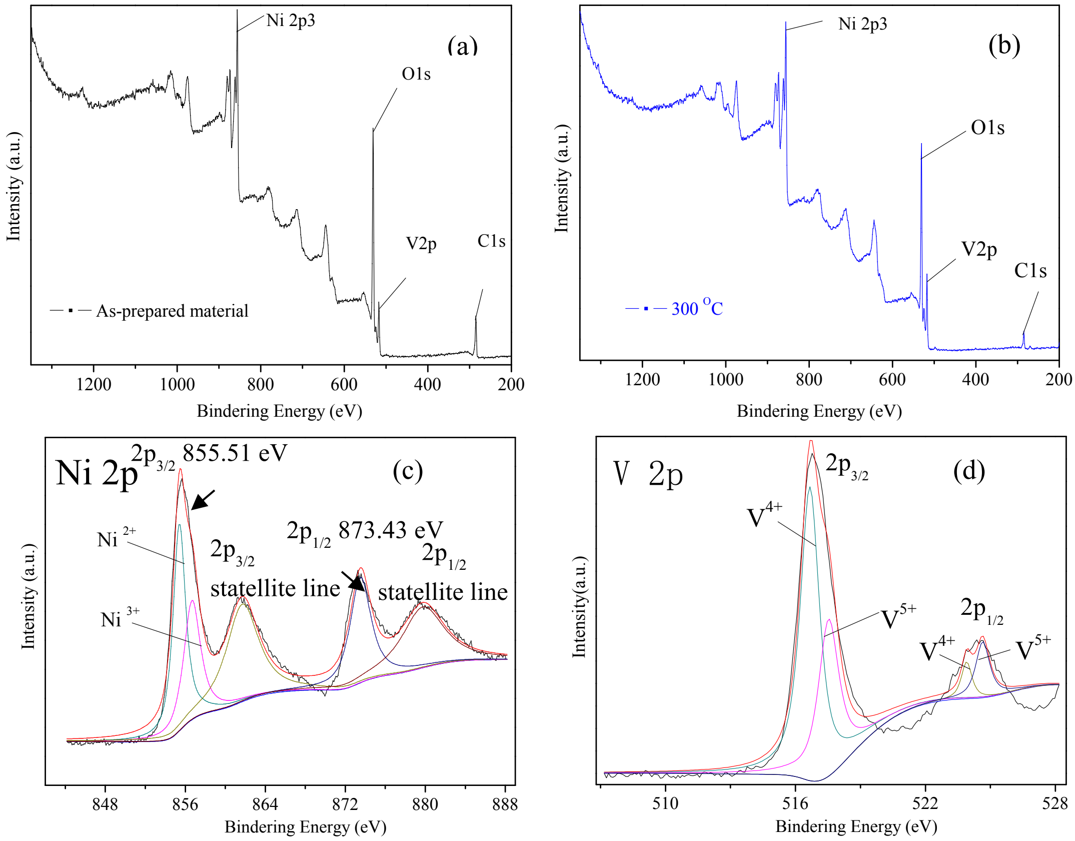


**Figure S2.** XPS spectra of survey spectrum from as-prepared $Ni_3V_2O_8$ nanowire arrays (a) and that after annealing at 300 °C (b); and the peak deconvolution and fittings of the Ni 2p (c) and V 2p (d).

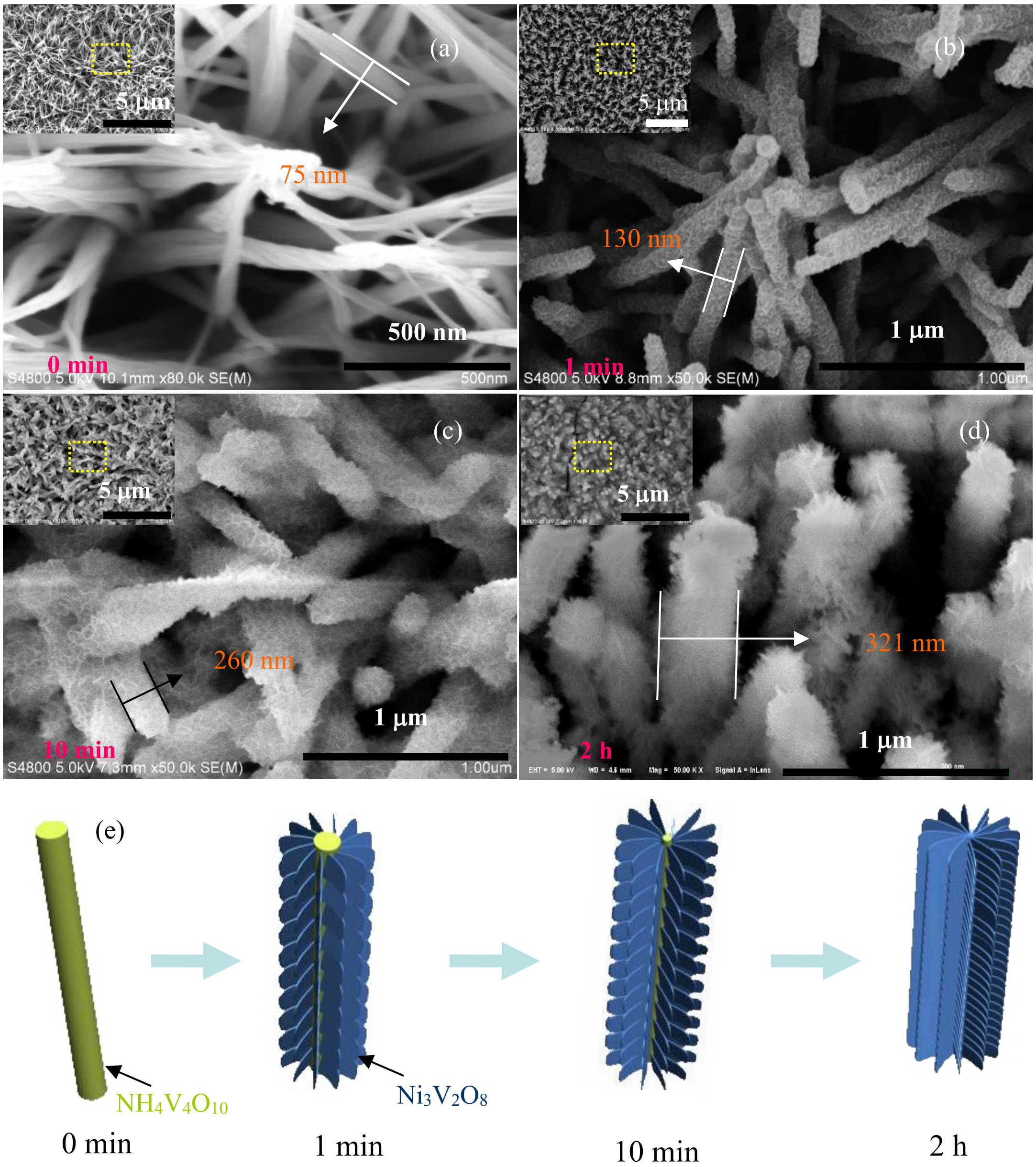


**Figure S3.** the samples obtained by a second hydrothermal reaction of vanadate ammonia and nickel salt with different reaction time: (a) 0 min, (b) 1 min, (b) 10 min and (b) 2 h.

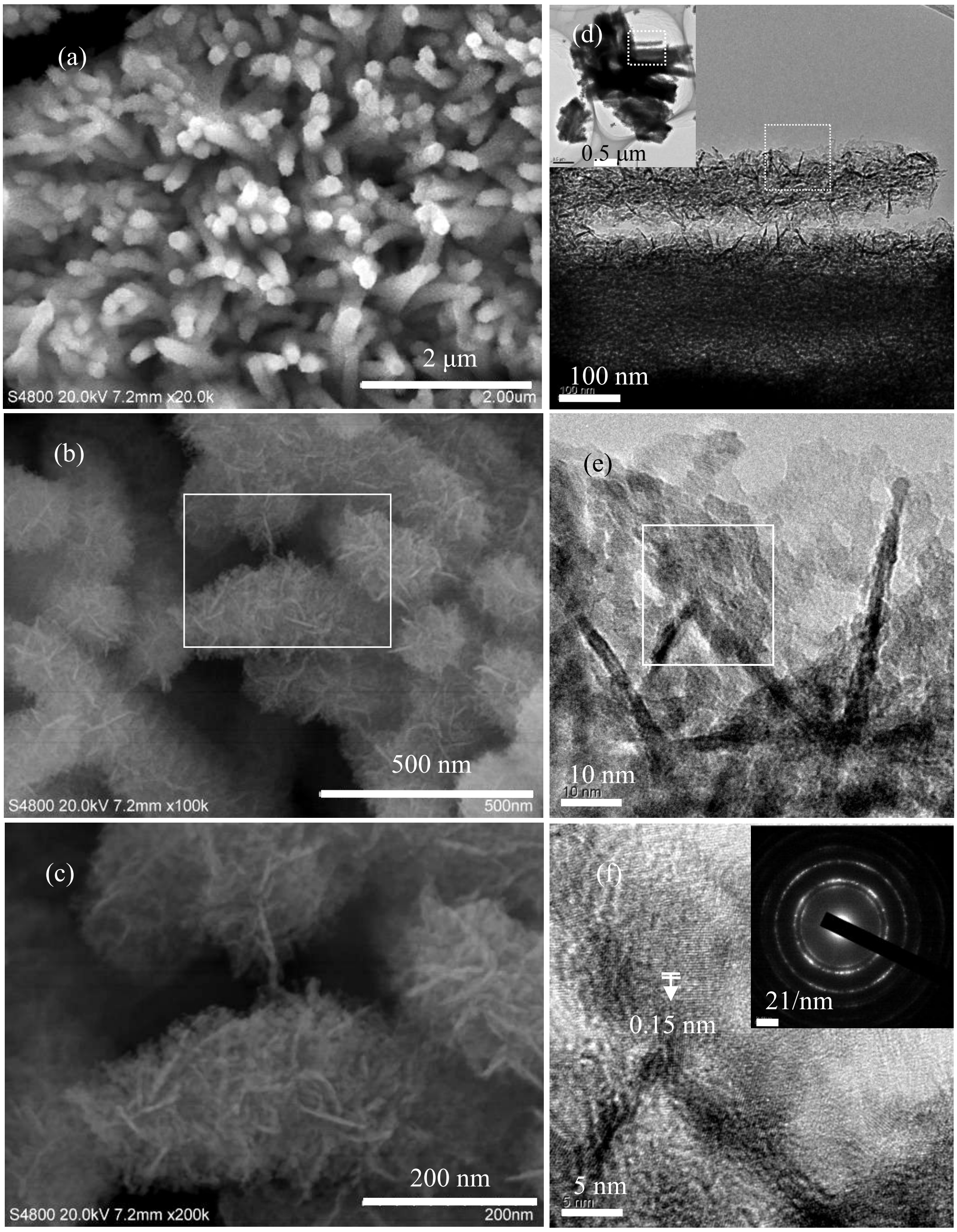


**Figure S4.** Typical FE-SEM images of the $Ni_3V_2O_8$ nanowire arrays with 300 °C heat treatment at different magnifications. Scale bars, (a) 2 μm; (b) 500 nm; (c) 200 nm; (d) and (e) TEM images of $Ni_3V_2O_8$ nanowire arrays (300 °C) detached from Ti foil, and (f) HR-TEM image (inset: SAED pattern).

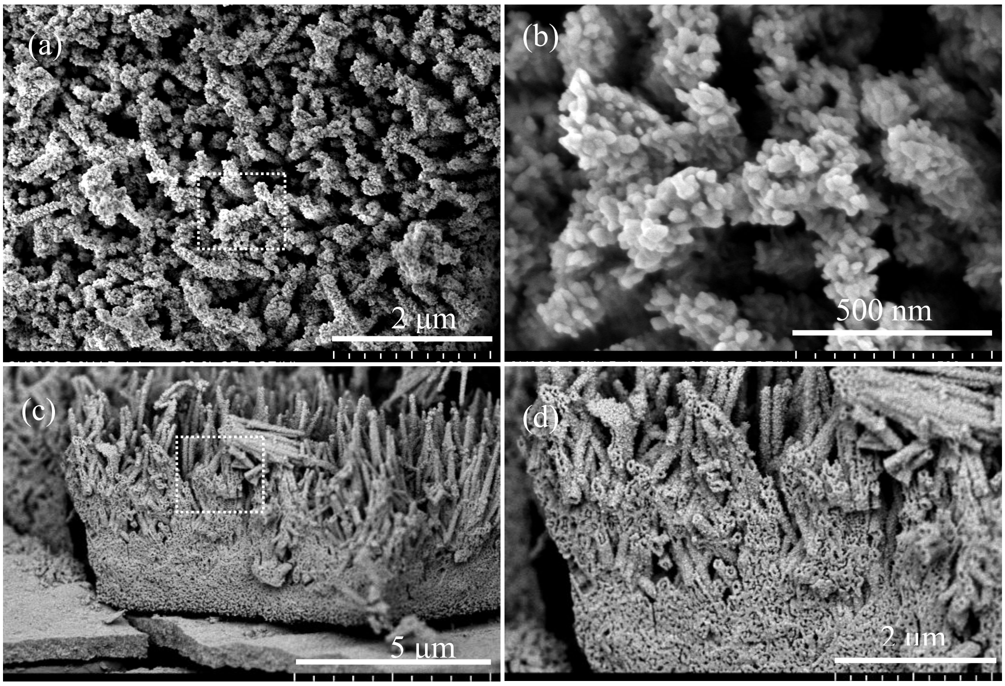


**Figure S5.** Typical FE-SEM images of the $Ni_3V_2O_8$ nanowire arrays growing on Ti foil with 500 °C heat treatment at different magnifications: a) and b) top-view; c) and d) cross-section view.

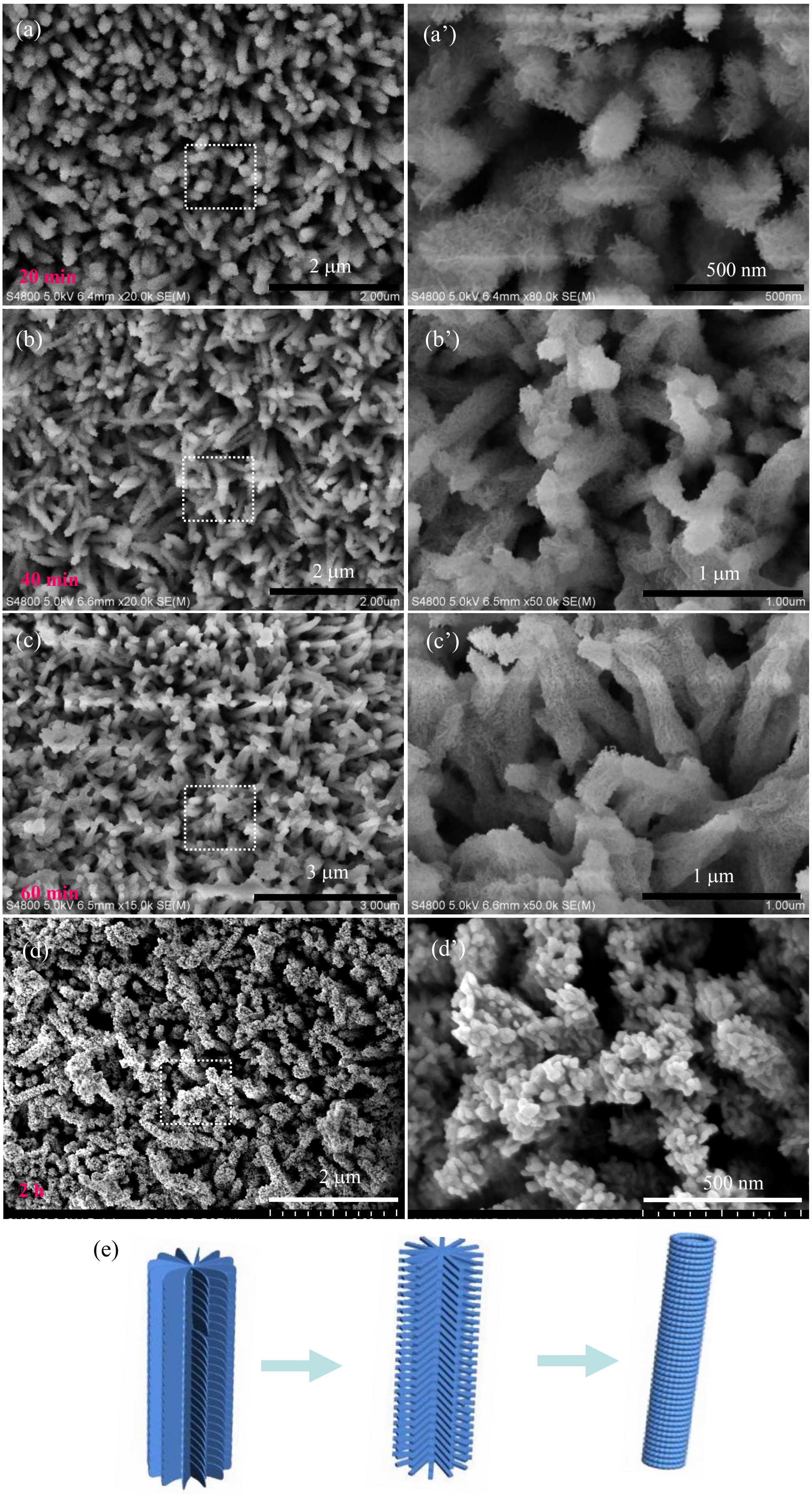


**Figure S6**. The morphology evolution of $Ni_3V_2O_8$ nanowire composed by nanosheets

with increasing the reaction time at 500 ºC.

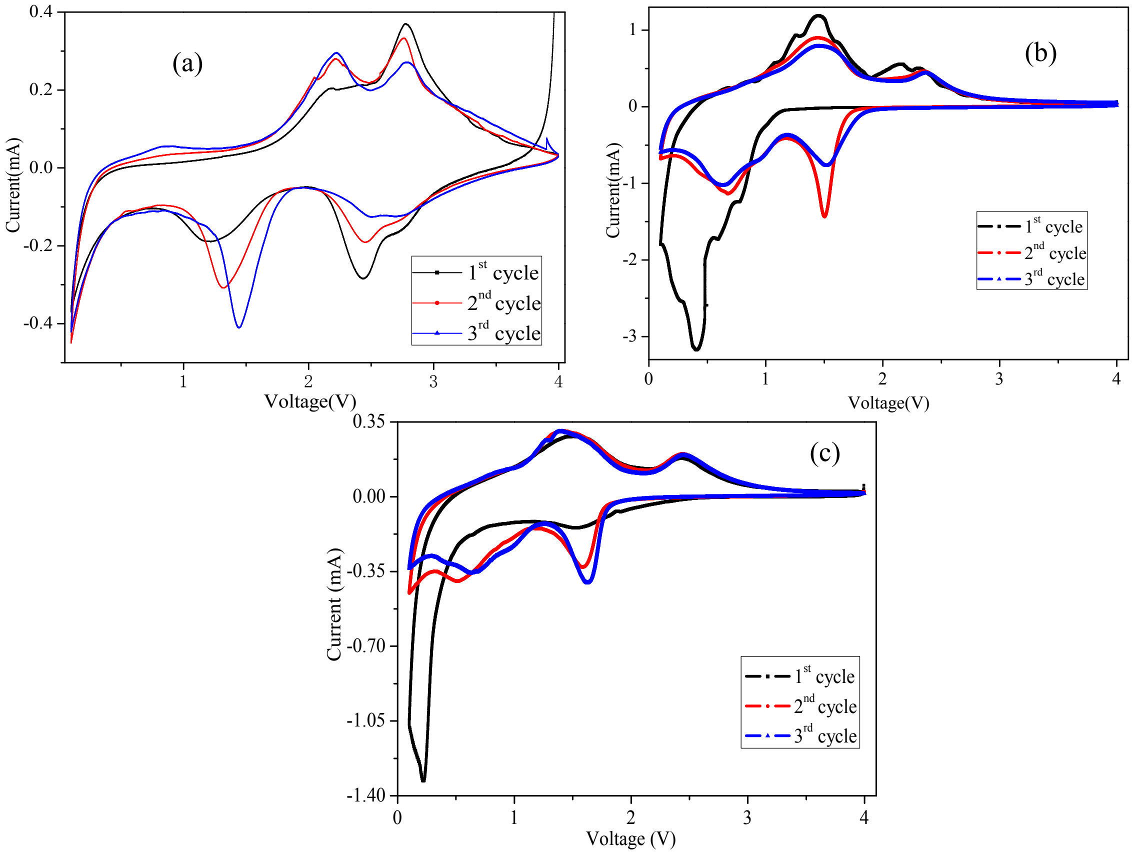


**Figure S7.** (a)-(c) show plots of the initial three the cyclic voltammogram (CV) curves at 0.1 mV·s$^{-1}$: (a) $NH_4V_4O_{10}$ nanowire arrays; (b) the as-prepared $Ni_3V_2O_8$ nanowire arrays; and (c) $Ni_3V_2O_8$ nanowire arrays after annealing at 300 °C.

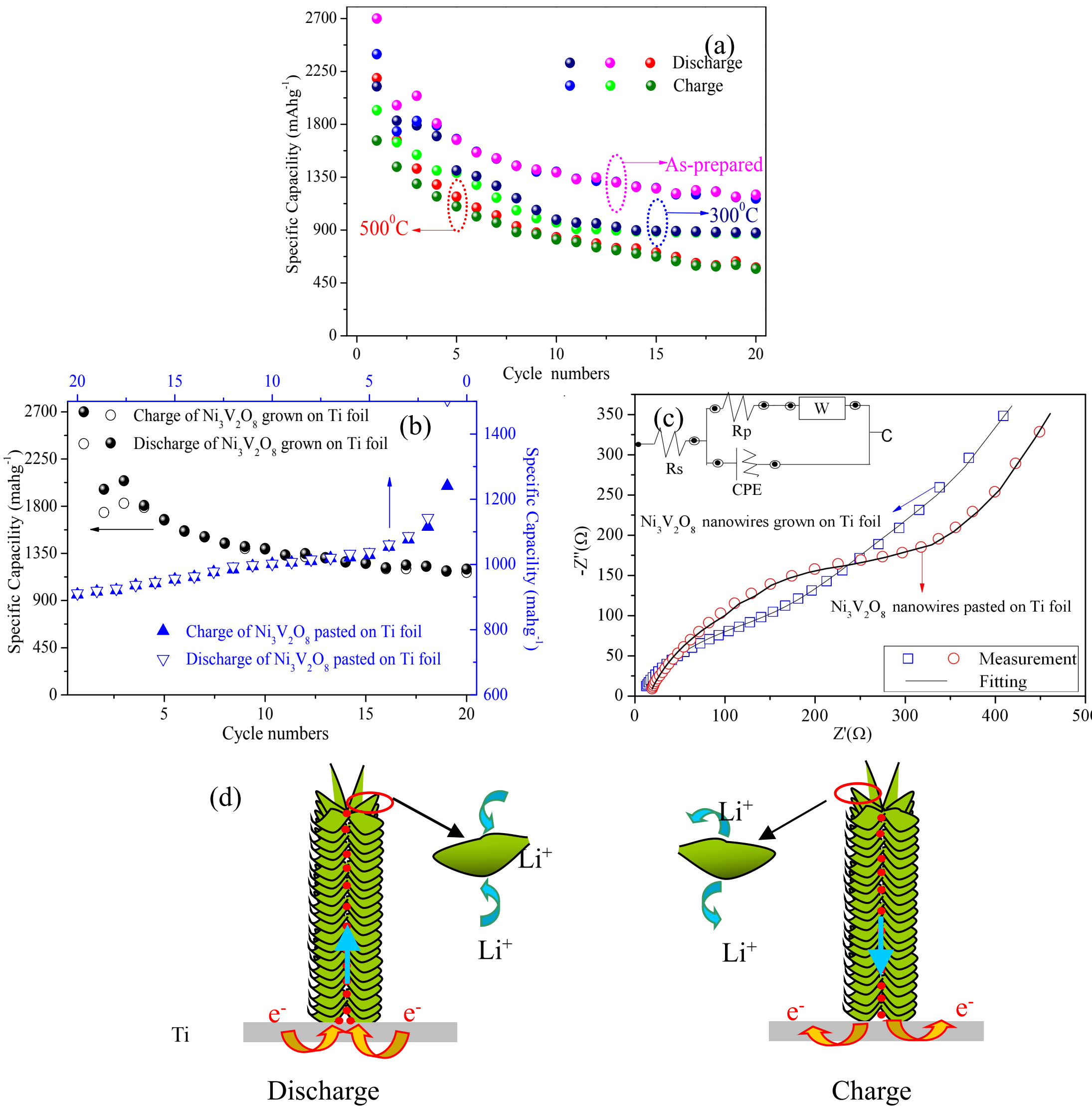


**Figure S8.** (a) Cycling performances of $Ni_3V_2O_8$ nanowire arrays electrodes calcinated at different temperatures at 300 mA·g$^{-1}$; (b) Cycling performances of $Ni_3V_2O_8$ nanowire arrays grown or pasted on the Ti foil at 300 mA·g$^{-1}$; (c) Nyquist plots of $Ni_3V_2O_8$ nanowire arrays in the frequency range from 5 kHz to 0.01 Hz, and the inset is the equivalent circuit; and (d) Schematic of charge storage of the $Ni_3V_2O_8$ nanowire array in LIBs.

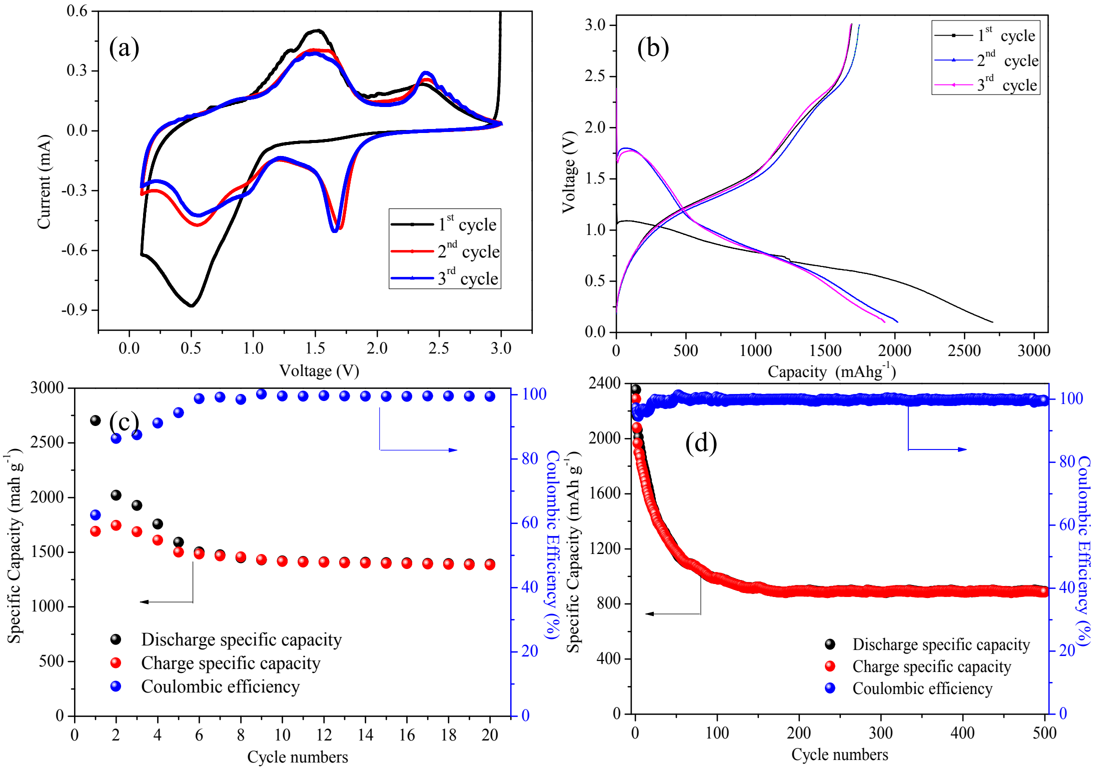


**Figure S9.** Electrochemical lithium storage properties of $Ni_3V_2O_8$ nanowire arrays grown on Ti foil between 3.0-0.1 V: (a) the CV curves at a scan rate of 0.1 $mV·s^{-1}$; (b) the initial three charge/discharge voltage profiles at a constant current density of 50 $mA·g^{-1}$; and the cycling performance at a current density of (c) 50 $mA·g^{-1}$ or (d) 500 $mA·g^{-1}$.

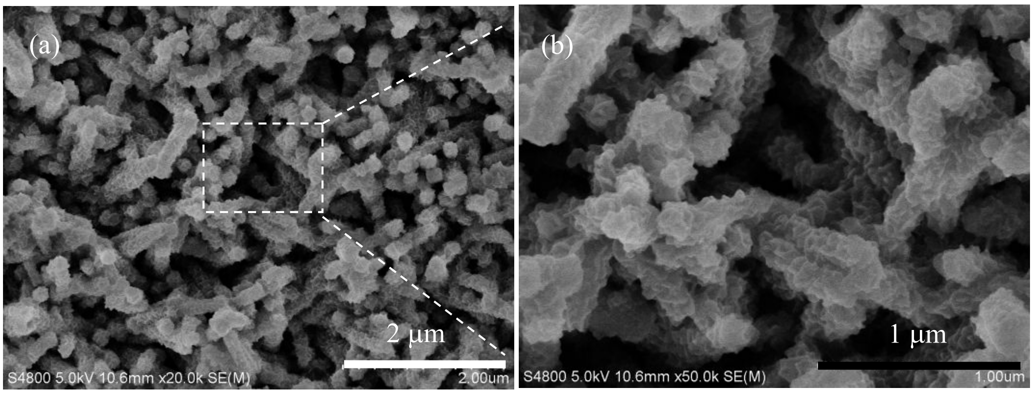


**Figure S10.** SEM images of the $Ni_3V_2O_8$ electrode after 50 cycles at a charge/discharge current of 500 mA $g^{-1}$.

**Table S1.** Structure parameters of $Ni_3V_2O_8$ nanowire arrays after annealing at different temperatures

| 2θ (°) | FWHM | Crystallite size (nm) | *d* space | Phase |
|---|---|---|---|---|
| | | Room Temperature (25 ºC) | | |
| 35.68 | 1.80 | 8.29 | 2.57 | 221 |
| 43.36 | 1.54 | 5.42 | 2.09 | 240 |
| 63.83 | 2.29 | 3.95 | 1.49 | 244 |
| | | 300 ºC | | |
| 35.82 | 1.54 | 8.77 | 2.50 | 221 |
| 43.94 | 1.12 | 7.10 | 2.07 | 240 |
| 63.87 | 1.41 | 6.50 | 1.46 | 244 |
| | | 500 ºC | | |
| 35.99 | 1.35 | 8.95 | 2.48 | 221 |
| 43.98 | 0.86 | 9.27 | 2.06 | 240 |
| 63.91 | 1.07 | 8.65 | 1.45 | 244 |

**Table S2.** Electrode resistances for $Ni_3V_2O_8$ nanowire arrays grown or pasted on Ti file obtained from equivalent circuit fitting of EIS results

| | $Ni_3V_2O_8$ nanowire arrays | $Ni_3V_2O_8$ nanowire powder |
|---|---|---|
| Rs (Ω) | 6.16 | 15.7 |
| Rp (Ω) | 160 | 272 |
| CPE(Yo) (μmh$_0$) | 18.7 | 24.6 |
| W (μmh$_0$) | 487 | 464 |

[1] P. Soudan, J.P. Pereira-Ramos, G. Gregoire, N. Baffier, The sol-gel mixed oxide $Cr_{0.11}V_2O_{5.16}$: An attractive cathodic material for secondary lithium batteries. Ionics, 1997, 3(3-4): 261-264.
